# How Do Middle-Aged Chinese Men and Women Balance Caregiving and Employment Income?

**DOI:** 10.3390/healthcare9040415

**Published:** 2021-04-03

**Authors:** Huamin Chai, Rui Fu, Peter C. Coyte

**Affiliations:** 1 School of Public Administration, East China Normal University, Shanghai 200241, China; huaminchai@126.com; 2 Center for Public Policy Research, East China Normal University, Shanghai 200241, China; 3 Institute of Health Policy, Management and Evaluation, Dalla Lana School of Public Health, University of Toronto, Toronto, ON M5T 3M6, Canada; peter.coyte@utoronto.ca

**Keywords:** informal caregiving, unpaid family caregivers, labour force participation, income, labour supply

## Abstract

Unpaid family caregivers might suffer losses in income as a result of care provision. Here we used data from the baseline survey of the China Health and Retirement Longitudinal Study to assess the relationship between hours of weekly caregiving provided to grandchildren/parents/parents-in-law and individual’s monthly employment income. Our study sample comprised 3718 middle-aged Chinese adults who were of working age (45–60 years). For women and men separately, we used a likelihood-based method to determine a caregiving threshold in a two-stage Heckman selection procedure. Instrumental variables were used to rule out the endogeneity of caregiving hours. Our analysis revealed a negative association between caregiving and income for women that depended on a caregiving threshold of 63 h per week. There was an absence of caregiving-income relationship among men. These results offer new insights into the opportunity costs of unpaid caregiving and support tailored policies to protect the financial well-being of female caregivers.

## 1. Introduction

### 1.1. Background

Asia is rapidly aging, which places enormous pressure on health systems, labour markets and the economy [1]. The limited supply of publicly funded eldercare results in high demand for unpaid home-based informal care provision, where adult children, especially daughters and daughters-in-law, are usually tasked with such responsibility [2,3,4,5]. At the other end of the spectrum, childcare in these countries also relies heavily on unpaid family caregivers, with grandparents often assuming the parental role for their grandchildren [6,7,8]. The deep-rooted filial piety and Confucian values further enhance individual duty to prioritise the well-being of elders and children in the family before their own career and personal advancements [9,10].

The most populous country in Asia and worldwide—China—is experiencing a striking decline in its working population that is projected to fall by 9% over the next two decades [11]. Working-age Chinese adults in the middle of their lives are commonly tasked with caregiving duties, in particular, to care for their elderly parents and grandchildren. Chinese law has mandated adults to provide care and financial support to elderly parents since 2012 [12], while statistics have shown at least 60% of Chinese grandparents are taking the primary caregiver role for grandchildren, a ratio much higher than most Asian countries (such as South Korea) [6]. As Chinese adults are increasingly expected to balance work and caregiving, it is important to understand if, and to what extent, allotting time to caregiving might reduce career prospects and employment income [13]. This is especially relevant after China’s recent announcement of a potential raise in retirement age [14]. As such, the purpose of this study is to identify the association between unpaid caregiving and the employment income of middle-aged Chinese adults who are of working age (i.e., between 45 and 60 years). These results are potentially useful for the design of policies that simultaneously promote family care provision, labour market attachment and welfare programs that safeguard the financial interest of working adults who are unpaid caregivers.

### 1.2. Literature Review

There are many studies in the extensive international literature that have documented the labour market consequences of engaging in unpaid caregiving [13]. There is overwhelming evidence to suggest that individual’s rate of labour force participation and working hours are inversely related to the intensity of caregiving, and that there might exist a caregiving threshold beyond which caregiving reduces these outcomes more than before that threshold [2,15,16,17,18,19,20,21].

Labour force participants’ employment income encompasses their total earnings gained from employment/self-employment; hence, it can be calculated as the product of hourly wages and hours worked. Research that directly assessed a relationship between unpaid caregiving and employment income has only been conducted on women providing care to parents/parents-in-law. A US study found women who were intensive caregivers (by providing at least 100 h of caregiving in the past 2 years) experienced a reduction in annual employment income in the long run [22], while a Chinese study suggested an absence of association between monthly employment income of women and any care provision or intensive caregiving using thresholds of 10, 15 or 20 h per week [2]. Other studies were conducted on hourly wages, i.e., how caregiving might reduce individual’s prospect of obtaining a high-paying job. Of seven studies on men [17,18,19,23,24,25,26], two of them—both conducted in the United Kingdom—found care provision reduced men’s wages [23,24]. Meanwhile, of 12 studies on women, only four studies offered conclusive findings of the absence of an association between caregiving and wages [18,20,25,26]. The remaining studies either found any care provision reduced women’s wages [19,24] or identified a 4%–18% decrease in wages only among those who were considered intensive caregivers using a weekly caregiving threshold of 10, 15 or 20 h per week [15,17,23,27,28].

Based on this review, we identified three major gaps in the literature concerning the relationship between unpaid caregiving and employment income: first, the definition of intensive caregivers varied across studies as the caregiving thresholds used were arbitrarily selected (e.g., 5, 10, 15 or 20 h of caregiving per week) rather than statistically determined. Second, the presence of a caregiving threshold could, in theory, lead to both kinks and/or discontinuities in the relationship between caregiving and income [29]; however, no study to date has statistically determined the exact form of that relationship, i.e., to simultaneously test for the presence, and to estimate the direction and magnitude, of such kinks and discontinuities. Third, findings from Western countries are unlikely to generalise to Asian countries due to intrinsic differences in the demographics, cultural drivers of family caregiving, as well as the settings and availability of public health care; hence, our study endeavors to address the current paucity of Asian studies on this topic [2,17]. Hence, we conducted this study to address these three research gaps.

In this population-based cross-sectional study, we used data from a large nationally representative sample of middle-aged Chinese adults. In order to assess a potentially non-linear association between individuals’ monthly employment income and weekly hours of unpaid caregiving, we followed the conceptual framework advanced by Van Houtven et al. 2019 [29] and statistically located a single caregiving threshold for women and men, respectively. Findings from our analysis are unique in the international literature and provide implications to decision-makers regarding health, social and labour policies.

## 2. Materials and Methods

### 2.1. Study Sample

We used person-level survey data from the first wave of the China Longitudinal Health and Retirement Study (CHARLS) that was conducted on a nationally representative cohort of Chinese adults aged 45 or above. The survey was developed using well-validated survey instruments from existing large-scale longitudinal studies on health and ageing, including the US-based Health and Retirement Study (HRS) and the Survey of Health, Aging and Retirement in Europe (SHARE) [30,31]. After the success of a pilot survey on two Chinese provinces in 2008–2009, the first national wave of CHARLS was launched in 2011–2012 [32]. Details on the sampling and recruitment procedures for the CHARLS can be found elsewhere [33]. These procedures yielded 10,257 households comprising 17,708 eligible individuals across 28 Chinese provinces. We chose to use the baseline CHARLS survey since it comprehensively captured data on individuals’ care provision, while the subsequent follow-up surveys (conducted in 2013 and 2015, respectively) that were available at the time of the present study did not collect these data. 

For the purpose of our analysis, we performed exclusions on the CHARLS cohort. First, we restricted the study sample to comprise only working-age adults since they were potentially able to earn employment income. This corresponds to men aged between 45 and 60 and women aged between 45 and 55 to align with the mandatory retirement age of 60 for men, 55 for white-collar women and 50 for blue-collar women in China [34]. These exclusions reduced the sample to 8603 (48.6% of the original sample) individuals for further consideration. We then excluded individuals who were either farm workers or unpaid family business workers (*N* = 4140) or reported to be self-employed by working in a family business (*N* = 264) because the income for these individuals were not reported. Furthermore, the labour supply of farm workers are commonly regarded to follow a differing pattern than that of non-farm workers [35]; hence, we restricted the scope of our analysis to be non-farm workers and reflected this in the study sample. Finally, we excluded individuals that were not confronted with a choice to balance caregiving and work due to not having a potential care recipient. In the CHARLS, participants were asked about their caregiving activities provided to a grandchild under the age 16 or an elderly parent (or parent-in-law) over the past year (see below); hence, we removed individuals who either did not have grandchildren under the age of 16 or did not have at least one parent (or parent-in-law) that was alive (*N* = 368) from the study sample. These selection procedures yielded 3718 individuals for our study sample, comprising 1450 women and 2268 men.

### 2.2. Dependent and Independent Variables

Individuals’ average monthly employment income over the past year was the primary outcome from our analysis. We first identified individual’s labour force participation status by coding labour force participants as 1 and non-participants to be 0. In the CHARLS, labour force non-participants were not asked about their employment income; hence, we considered a value 0 for their monthly employment income. For labour force participants, we calculated their past-year average monthly employment income as the sum of their monthly salary and bonus after tax [2]. In the regression analyses, monthly employment income was log-transformed to correct for skewness [36]. 

We assessed individuals’ weekly hours of unpaid caregiving offered to grandchildren under the age 16 and/or parents/parents-in-law over the past year as the main independent variable. The CHARLS has used the age 16 to define grandchildren that are likely to depend on the care of grandparents, while the care provided to those above 16 was not measured [35,37,38]. Care work was defined to be any assistance in daily activities (ADLs) or other activities, including but not limited to household chores, meal preparation, laundry, going out, grocery shopping and financial management [38]. Individuals who did not report any caregiving activities were termed “non-caregivers” and assigned a value of 0 for their weekly caregiving hours. The remaining individuals’ (i.e., caregivers) weekly caregiving hours were computed as the sum of total weekly hours of childcare (provided to grandchildren) plus that of eldercare (provided to parents/parents-in-law). Secondary analyses that separately examined the hours of care provided to elders (parents and/or parents-in-law) and to grandchildren were also conducted (see below). 

Other independent variables considered in the analysis were based on findings from previous literature [13,15,19,20]: age and age-squared; marital status (currently married or unmarried); highest education (illiterate or primary school, middle school, high school, or college and above); residential location (rural or urban); household size (the number of people living in a household); living with parents and/or parents-in-law (yes or no); having an outpatient visit in the last month or an inpatient admission in the last year (yes or no); household yearly income from all sources (log-transformed); whether individuals worked at a government or state-owned organisation or firm; and whether individuals held a position as a manager.

### 2.3. Statistical Analysis

#### 2.3.1. Basic Model Setup

We conducted separate regression analyses on women and men to reflect their differential opportunity costs of caregiving [23]. We specified a log-linear model and used three caregiving variables to predict monthly employment income: a continuous variable denoting weekly hours of caregiving (CG); an indicator variable denoting a discontinuity in income once caregiving reached or exceeded an unknown threshold, T (CG^ = 1 if CG ≥ T, otherwise CG^ = 0); and an interaction term of the two previous variables representing a potential kink in the caregiving-income association once caregiving exceeded the same threshold (CG*CG^) [29]. The threshold T was located by testing a range of values between 0 and 140 h of caregiving per week with increments of 1–10 h depending on the availability of observations. The value that maximised the likelihood function of the corresponding log-linear model was selected. Using this model, we performed two joint significance tests to verify the presence of an overall association between caregiving and income (i.e., joint significance of the coefficients of CG, CG^ and CG*CG^) and to test for a significant change in the association between caregiving and income due to the presence of the identified caregiving threshold (i.e., joint significance of the coefficients of CG^ and CG*CG^).

#### 2.3.2. Addressing the Potential Selection Bias and Endogeneity of Caregiving Hours

Using this basic model setup, we investigated selection bias and the possibility that caregiving hours was an endogenous regressor. In the CHARLS, respondents who self-identified to be labour force non-participants were censored on questions regarding income. This raised potential selection bias if we were to exclude all labour force non-participants from the income analysis. Hence, a two-step Heckman selection modelling procedure was performed [39]. In the first step, a probit model was estimated for all survey respondents to predict whether they were labour force participants (yes/no) using all observed covariates except for whether individuals were employed at a government or state-owned organisation or firm and whether individuals held a position as a manager (since both variables were also censored for labour force non-participants). An inverse Mills Ratio (IMR) that was computed from the probit model was entered into the log-linear model to predict monthly employment income with the three caregiving variables and all observed covariates. The significance of the IMR in the log-linear model would signal the presence of selection bias in our analysis.

In order to rule out the endogeneity of caregiving hours, an instrumental variables (IV) method was used. In principle, there are two reasons to be concerned about the possible endogeneity of caregiving hours: first, there may be a reverse causal relationship between caregiving and employment income, and second, there may be a mutual set of determinants for the two. Several empirical analyses have corroborated such theoretical predictions by showing the endogeneity of caregiving [4,19,40,41,42]. To address this concern, we used four IVs, including: the presence of grandchildren under the age of 16 in the household; the number of these young grandchildren; if one of the eldercare recipients was in poor health; and the number of eldercare facilities in the community [38]. These IVs were chosen purposefully to be strong correlates of caregiving hours while being unrelated to monthly employment income [19,40,41,42,43,44]. Specifically, we expect middle-aged Chinese adults with more grandchildren to have more extensive involvement in grandchild care [6]; analogously, having an elderly parent or parent-in-law in poor health increases the propensity of Chinese adults to allocate more time to caregiving. The presence of an eldercare facility (such as a public nursing home) in the community has been shown to substitute for unpaid eldercare provided by adult children [25,45]; hence, we expect Chinese adults who have access to such public establishments tend to provide less caregiving to parents/parents-in-law [35]. Validity of the four IVs was assessed through three routinely performed statistical tests, including the tests of under-identification, over-identification and weak identification [46,47,48]. Using these IVs, we performed a Limited-Information Maximum Likelihood (LIML) procedure [49,50] rather than the commonly implemented Two-Stage Least-Squares to mitigate the potential bias caused by weak IVs [51]. In this procedure, two regression equations were simultaneously estimated: a linear regression predicting weekly hours of caregiving using the four IVs and all observed covariates; and a log-linear regression predicting monthly employment income with the three caregiving variables, the same set of covariates and the inverse Mills ratio. An endogeneity test was performed to assess the presence of endogenous bias [46,47,52,53]. The Heckman selection model without the use of the four IVs would be deemed the preferred model if we were unable to reject the exogeneity of caregiving hours using a 2-sided *p*-value of 0.05.

#### 2.3.3. Secondary and Sensitivity Analyses

Two sets of secondary analyses were conducted. We first repeated the Heckman selection procedure and the IV analysis separately using hours of eldercare (provided to one’s parents and/or parent-in-law) and childcare (provided to one’s grandchildren). Then, the analysis was conducted on a pooled sample comprising both women and men. A dummy variable indicating the gender of the participants (male vs. female) was entered into the regression equations. We used the same likelihood-based method to locate a new weekly caregiving hours threshold in each iteration. 

We conducted two sets of sensitivity analysis. First, in order to demonstrate the merit of representing caregiving hours using three variables (i.e., CG, CG^ and CG*CG^), we compared the statistical performance of our comprehensive model (i.e., either the Heckman selection model without the IVs or the IV model) with four simpler models that had been previously proposed in the literature [18,19,20]. These alternative models represented caregiving by using: (1) a single indicator variable that distinguished caregivers from non-caregivers; (2) a single continuous variable reflecting the weekly hours of caregiving (CG); (3) a single indicator variable representing if individual’s weekly caregiving hours were above the identified threshold (CG^); (4) an interaction term between the threshold dummy variable and hours of caregiving to indicate a kink (i.e., change of the slope) in the association between caregiving and income after hours of caregiving exceeded the threshold (CG*CG^). We used the coefficient of determination and pairwise likelihood ratio tests to compare each model with our main model. 

Next, we undertook seven sets of subgroup analysis to assess if specific individual characteristics impacted the identified caregiving-income relationship. These analyses were conducted using the comprehensive model and assumed the same caregiving threshold identified from the primary analysis. We considered whether participants: lived with their parents and/or parents-in-law (yes vs. no); their types of employment (employed vs. self-employed); types of hukou or household registration status (rural hukou vs. urban hukou); education attainment (below middle school vs. middle school or above); household income (below the median level vs. equal to or above the median level); hukou region (in the east vs. middle vs. west Chinese economic macro-region); and the city tier where participants had their hukou registered (cities in a Tier 2 or above vs. cities below Tier 2) [54].

## 3. Results

### 3.1. Descriptive Analysis

Table 1 takes a preliminary look at the distribution of unpaid weekly caregiving hours, labour force participation (LFP) and monthly employment income among women and men in our sample. Among 1274 women, 41% (*N* = 524) were caregivers. In general, women who were caregivers had lower LFP rates and employment income compared to their non-caregiving counterparts. Among women caregivers, their LFP rates and employment income tended to decrease consistently with an increase in weekly caregiving hours. Furthermore, women who only provided care to grandchildren (*N* = 249, 48% of all caregiving women) reported the lowest LFP rates and employment income compared to women who either only provided eldercare or cared for both dependents. Similar findings can be drawn for men.

Among labour force participants, we reported their characteristics stratified by gender and caregiving status in Table 2. For labour force–participating women (*N* = 1274), those who were caregivers over the past year were slightly older (mean age = 49.15 vs. 48.44 years, *p*-value < 0.01); lived in a larger household (having an average of 3.78 vs. 3.39 persons, *p*-value < 0.01); were more likely to have had either an outpatient visit in the past month or an inpatient admission in the past year (25% vs. 15%, *p*-value < 0.01); and earned ¥495 less on average per month (*p*-value < 0.01). Similarly, among labour force–participating men (*N* = 2053), those who also provided unpaid caregiving were older (mean age = 52.06 vs. 51.34 years, *p*-value < 0.01); lived in a larger household (having an average of 4.09 vs. 3.71 persons, *p*-value < 0.01); and had lower employment income (mean monthly employment income = ¥2386.41 vs. ¥2720.61, *p*-value < 0.05). Moreover, labour force–participating men who were caregivers were more likely to be college graduates (7% vs. 5%, *p*-value < 0.05); lived with their parents (20% vs. 15%, *p*-value < 0.05); and worked at a government or state-owned organisation or firm (27% vs. 23%, *p*-value < 0.05).

### 3.2. Endogeneity of Weekly Caregiving Hours

Using the four instrumental variables (IVs), we ruled out the endogeneity of weekly caregiving hours for both women and men (both *p*-values > 0.1 in the endogeneity test). Hence, we considered the Heckman selection model without the use of IVs to be preferred and proceeded with the analysis using this model. Results of the IV analysis are reported in Table A1 in Appendix A. In terms of the validity of the chosen IVs, for both women and men the IVs were deemed weak (Kleibergen-Paap rk Wald F-statistic = 0.68 and 1.63 for women and men, respectively) and passed the overidentification test (both *p*-values > 0.1). For men, we successfully rejected the null hypothesis of under-identification (*p*-value = 0.04), but for women, the IVs failed the test of under-identification (*p*-value = 0.3). 

### 3.3. Caregiving-Income Relationship among Women and Men

A caregiving threshold of 63 h per week was identified for women (Table 3). Overall, we observed a significant negative association between women’s weekly caregiving hours and monthly employment income (joint significance of the three caregiving hours variables <0.01), and that this association depended on a caregiving threshold of 63-h per week (joint significance of the discontinuity and interaction <0.05). We also found strong evidence of selection bias as labour force–participating women tended to earn higher employment income than did their non-participating counterparts (*p*-value of the inverse Mills ratio <0.05). However, the coefficients of the three caregiving variables were individually insignificant (all *p*-values > 0.05), indicating a lack of association between caregiving and employment income on either side of the 63-h caregiving threshold and an absence of an income discontinuity at the threshold.

Other independent correlates of higher monthly employment income among women were: unmarried (*p*-value < 0.05); college graduates as opposed to being illiterate or having only completed primary school (*p*-value < 0.05); living with parents/parents-in-law (*p*-value < 0.05); having a smaller household (*p*-value < 0.05); higher household income (*p*-value < 0.01); working as a manager (*p*-value < 0.01); not working at a government or state-owned organisation or firm (*p*-value < 0.05); and not having any outpatient visits in the last month or being hospitalised over the last year (*p*-value < 0.05).

For men, our analysis identified 15 caregiving hours per week as a threshold (Table 3). There was an absence of an overall association between weekly caregiving hours and monthly employment income among men (joint significance of the three caregiving hours variables >0.05) nor did the caregiving-income association changed after men have reached or exceeded the 15-h threshold (joint significance of the discontinuity and interaction >0.1). We did identify the presence of selection bias as men who were labour force participants reported greater employment income (*p*-value of the inverse Mills ratio <0.01). Since none of the three caregiving variables were individually significant (all *p*-values > 0.1), we conducted sensitivity analysis to explore alternative models that excluded the caregiving threshold (see below). 

Other independent correlates of higher monthly employment income among men were being married (*p*-value < 0.01); high school (*p*-value < 0.05) or college graduates (*p*-value < 0.01); rural rather than urban residents (*p*-value < 0.01); living in smaller household (*p*-value < 0.01); higher household income (*p*-value < 0.01); working as a manager (*p*-value < 0.01); and not having any past-month outpatient visits or past-year inpatient stay (*p*-value < 0.01).

### 3.4. Secondary Analysis

Table 4 summarises results of the first secondary analysis where separate Heckman selection modelling procedures were conducted on eldercare and childcare. For women, we identified 63 h and 72 h to be the respective thresholds for weekly hours for eldercare and childcare. For both types of care, there was a significant overall negative association with employment income (both *p*-values < 0.05), but the threshold effect was only significant in eldercare (*p*-value < 0.01). Specifically, the negative eldercare-income relationship was driven solely by a sudden decrease of employment income at the 63-h eldercare threshold (*p*-value < 0.05) since this relationship was insignificant on either side of that threshold. With regard to childcare, we found that women’s employment income started to decrease immediately as time was allotted to caring for grandchildren (*p*-value < 0.01) with no discontinuity of income at the 72-h threshold or any change in the slope thereafter. For men, we identified 15 h and 10 h to be the weekly thresholds for eldercare and childcare, respectively. While the results on both types of care were largely similar to our primary findings, we did identify an overall association of men’s employment income with their hours of childcare (*p*-value < 0.01). For these analyses, we also used the four IVs and found caregiving hours to be exogeneous on all occasions (see Table A2 ). 

Table A3 and Table A4 in Appendix A present the results of the Heckman analysis without or with the four IVs conducted on the pooled sample of women and men. Because we failed to reject the exogeneity of caregiving (*p*-value = 0.31 in the endogeneity test), results of the Heckman model without the use of IVs (Table A3) were used to demonstrate the relationship between caregiving and employment income for the pooled sample. We located 72 h of caregiving per week to be an important threshold, where an overall negative association between caregiving and employment income was confirmed (*p*-value < 0.001) and the threshold effect was ruled out (*p*-value = 0.12). For both women and men, we estimated their monthly employment income to decrease immediately as time was spent on caregiving (*p*-value < 0.01), without experiencing any further discontinuities or kinks in this relationship (both *p*-values > 0.05). Moreover, we found men to earn a significantly higher level of employment income than women (*p*-value < 0.01).

### 3.5. Sensitivity Analysis

Table 5 reports the results of using alternative model specifications to predict log-transformed monthly employment income for women and men. For women, we found evidence that our model outperformed the model that only treated caregiving as a dummy variable (distinguishing caregivers and non-caregivers), the model where caregiving hours were represented by only a single continuous variable, and the kink-only model (all *p*-values of pairwise likelihood ratio tests < 0.05). However, we failed to detect any meaningful improvement of our model over the discontinuity-only model (*p*-value > 0.1). In terms of the coefficient of determination, it was maximised in our model and the discontinuity-only model (in both cases R-squared = 0.169) while being much smaller in other models. For men, our model was not significantly better when compared to all of the four simpler models (all *p*-values of pairwise likelihood ratio tests > 0.1). However, the R-squared was improved in our model and the discontinuity-only model (in both cases R-squared = 0.196), thereby providing some support to the superiority of our model.

Next, using a caregiving threshold of 63 h and 15 h per week for women and men, respectively, we conducted subgroup analyses and reported the results in Table A5 and Table A6 in Appendix A. For women, we reached similar results regarding an income-caregiving relationship for the following subgroups of women: those who were not living with their parents; were employed rather than self-employed; had urban hukou; did not finish middle school; were living in a household with income below the median value; or had their hukou registered in a city ranked below Tier 2. For these women, there was an overall negative association between monthly employment income and weekly caregiving hours with the association being depended upon the 63-h caregiving threshold. For other women, we either did not identify the presence of an income-caregiving relationship at all or failed to detect a threshold effect. Notably, an incremental effect of caregiving on women’s employment income was only found significant among those with at least middle school education; their income started to decrease immediately given time spent on caregiving (*p*-value < 0.001) with a significant downward discontinuity of income at the 63-h caregiving threshold (*p*-value < 0.05). For men, we found the association between caregiving and employment income to be mostly absent, except for men with rural hukou (*p*-value < 0.05). For men with rural hukou, we found an overall positive relationship between monthly employment income and weekly caregiving hours, although none of the three caregiving variables were individually significant.

## 4. Discussion

### 4.1. Main Findings

An association between unpaid caregiving and an individual’s employment income has important implications for health and labour policies, especially in times of rapid population ageing [55]. In this study, we used data from a nationally representative sample of middle-aged Chinese adults to investigate a caregiving-income relationship based on gender and a potential caregiving threshold. We found an absence of such association among men and a negative association between employment income and caregiving for women that depended on a caregiving threshold of 63 h per week. 

In the primary analysis, we reached the conclusion that men’s employment income was unrelated to their hours of caregiving. To the best of our knowledge, this is a new finding in the international literature since no previous study has explicitly examined men’s caregiving-income association by using employment income as the outcome variable. Among studies that assessed men’s hourly wages, our results are in line with the findings from the majority of these studies [17,18,19,25,26]. In contrast, two early studies conducted in the United Kingdom suggested caregiving activities lowered men’s hourly wages. Carmichael and Charles [23] found British men who were intensive caregivers, i.e., those providing more than 10 h of unpaid care per week, earned 18% lower wages than those who were non-caregivers. Heitmueller and Inglis [24] advanced the same conclusion by showing British men who were any caregivers—regardless of caregiving intensity—expected lower wages than did otherwise identical non-caregiving men. We argue that these findings are not necessarily inconsistent with ours since we investigated men’s total monthly employment income in the analysis, which is based on the product between their hourly wage and the number of hours worked. Following the wage penalty conclusion drawn by these two studies, our results suggest that men in low-wage jobs tend to work longer hours to compensate for the reduction in total employment income. In the secondary analysis, we demonstrated that when restricting the type of caregiving to grandchild care, there was evidence of a negative association between men’s employment income and hours of caregiving. However, neither the discontinuity at the threshold of 15-h childcare nor the slope on either side of this threshold were found significant. We also considered the possibility of excluding the caregiving threshold in the sensitivity analysis, and thereby representing caregiving hours as a single continuous variable. While this more parsimonious model did find a significant incremental decrease in men’s employment income by caregiving hours, the magnitude was small—i.e., the estimated coefficient of CG = −0.00297 corresponding to a 0.3% decrease in men’s monthly employment income with an increase in one hour of weekly caregiving. Considering that the mean annual employment income of Chinese working men aged between 45 and 60 is ¥2619 from our sample, an hourly increase in weekly caregiving is associated with an average of ¥94 (or US$15) decrease in annual employment income. These findings warrant additional studies to comprehensively examine the simultaneous impact that care provision might have on men’s wage rates and labour supply, and if the type of caregiving plays a role in the determination of the caregiving-income relationship among men. 

At least two studies have explored the association between hours of eldercare (provided to parents/parents-in-law) and the employment income of women [2,22]. Using data from the US Health and Retirement Study, Fahle and McGarry found that women who provided at least 100 h of eldercare over the past 2 years were subject to a reduction in annual employment income over the next 2–10 years [22]. These findings are consistent with our identification of a decreasing trend in women’s employment income with more intensive eldercare provision. However, a Chinese study that shared a similar study design with ours found that the monthly employment income of Chinese married women was unrelated to eldercare, including any eldercare activity or intensive eldercare beyond 10, 15 or 20 h per week [2]. We offer two explanations for these inconsistent findings: first, while Chen et al. considered a cohort of young married women (aged 18–44 years), we studied older women (aged 45–60 years) where 6% of those women were currently unmarried. Hence, it is possible that the caregiving-income dynamics of older Chinese women is fundamentally different from that of their younger counterparts. Second, our conclusion was based on a statistically determined eldercare threshold of 63 h per week, which was larger than the arbitrarily chosen thresholds considered in their analysis. We argue that by not employing a statistically plausible method to locate a caregiving threshold, Chen et al. might have missed an opportunity to identify an overall inverse association between women’s employment income and hours of eldercare. Compared to previous studies that assessed women’s hourly wages and caregiving, our findings are in line with the majority of these studies as they found a wage penalty associated with intensive caregiving [15,17,19,23,24,27,28,56]. Regarding the remaining studies that did not suggest caregiving to influence women’s hourly wages [18,20,25,26], we argue that the discrepancy might have arisen from differed analytical settings (as these studies were based in either European or North American countries).

A novelty of our analysis was to study an exclusive association between women’s monthly employment income and weekly hours of childcare dedicated to grandchildren. We found women started to suffer losses in employment income once time was allocated to childcare; specifically, each hourly increase in childcare per week was associated with a 2% reduction in monthly employment income. These findings are unique in the international literature as previous studies have not isolated the hours of grandchild care from other types of caregiving. It is worth noting that although we located a grandchild care threshold of 72 h per week, there was no evidence of any threshold effect, which ruled out any kinks or discontinuities in how women’s employment income was associated with the hours of childcare. Hence, we conclude that women’s employment income reduced immediately and consistently with each incremental increase in the hours of childcare, which is a different pattern in the case of eldercare where an incremental effect was absent.

A large body of Asian literature has given compelling evidence of a positive association between co-residence with parents/parents-in-law on women’s labour force participation and working hours [8,57,58,59,60]. We advanced these findings by suggesting that the monthly employment income of women was also potentially enhanced by living with parents/parents-in-law by about 55%. Another finding of ours pertained to the marriage penalty on women’s employment income, as we found married women experienced a 53% reduction in monthly employment income compared to their unmarried counterparts, while men appeared to have doubled their employment income in marriage. This female marriage penalty has been repeatedly shown in the empirical literature [61,62,63,64,65,66] while having its theoretical root in Becker’s specialisation hypothesis [67] and the theory of the sexual division of labour [68]. It is worth noting that the employment income reduction in married women found by us was greater in magnitude compared to previous estimations, possibly due to our lack of adjustment for other family characteristics such as the number of children and siblings of the mother [25].

### 4.2. Policy Implications

Our findings offered important insights on policy interventions to assist in balancing the economic growth and sustainable supports for an ageing Asian population. At an employer level, family-friendly policies at the workplace need to be implemented to support workers who are also caring for an elderly parent (including parent-in-law) or a young grandchild. These include more flexible work hours, alternative work arrangements (such as job-sharing) and the development of training programs that help workers who are caregivers gain caregiving and time management skills. Extra institutional supports need to be made available to workers who are experiencing exceptional caregiving burden such as those providing palliative care to elderly parents or in-laws towards the end of life [69]. Furthermore, as our findings suggest caregiving might place greater income penalty on women than on men, employers should design policies to reflect such gendered difference by prioritising efforts to help female employees balance working and caregiving. On a broader scale, labour policy makers need to launch programs and laws to promote informal caregiving without discouraging caregivers’ commitment to the labour market. Such measures include higher tax credits to caregivers and more paid leaves and job-protection when associated with care provision.

Health policy makers need to recognise the vital role of unpaid caregivers and consider the potential to invest in targeted publicly financed formal care, especially services that substitute for unpaid caregiving, so that caregivers could potentially redirect time to labour market activities. Home-based formal care, particularly care provided by personal support workers, have been shown to substitute unpaid informal care [70,71]; hence, the investment on such services needs to be prioritised. Furthermore, fiscal support for the provision of home-based formal care may best be targeted to individuals requiring more intensive caregiving, as our findings suggest intensive caregivers are at the greatest risk of lower employment income due to their lower propensity to be in the labour force, and when in the labour force, tend to earn less employment income. Finally, our results call for the launch of publicly subsidised childcare programs including residential childcare, nursery schools and daycare centers to help relieve the pressure on grandparents. Such care establishments are nascent in many Asian countries including Korea and Japan that are more economically advanced [72,73,74].

### 4.3. Study Limitations

Findings of our analysis need to be interpreted with a few limitations in mind. First, we did not have access to data about whether individuals had cared for their spouse, their own children or other disabled or sick members of the family. However, grandchild care and parental care are the two most prevalent types of unpaid care tasks among Chinese adults between 45 and 60 years [75]; therefore, we believe that we have provided a relatively comprehensive analysis on the income-caregiving association in middle-aged Chinese adults. Future studies need to extend our analysis by examining individuals responsible for taking care of other family members to reveal the consequences of caregiving on their employment income. Second, we did not have information on whether individuals shared caregiving responsibilities with a spouse or other household members. Thus, researchers that design surveys for Chinese adults need to include items of primary/secondary caregiving and co-caregiving responsibilities to address this limitation of our analysis. Third, care dependents’ utilisation of publicly funded formal care was not documented, although the supply of such care was extremely limited in China at the time of the survey [76]. Following the launch of a pilot program in public long-term care insurance in 2016 [77], future studies with access to claims data detailing the use of such public services may yield additional insights regarding how individuals simultaneously decide on the utilisation of public care, their own care provision and paid work [78]. Finally, the cross-sectional data impeded our ability to assess the dynamic transition of care roles over time [27] or a longitudinal trend of caregiving and work in the population [22,24,26,28]. Future researchers that are interested in using the CHARLS data may consider linking multiple waves of the survey to allow for such panel data analyses.

## 5. Conclusions

In conclusion, we provided statistical evidence on a decreasing association between the monthly employment income of women with hours of weekly unpaid caregiving, while such association was demonstrated to be absent for men. These findings call for an array of policies and compensation programs to offer direct financial relief to female caregivers to help promote their labour market activities. Health policymakers need to explore options of long-term care reforms and other publicly funded substitutes for informal unpaid caregiving to ease the pressure on workers who are tasked with balancing caregiving and work.

## Figures and Tables

**Table 1:** Descriptive analysis on caregiving, labour force participation and monthly employment income.

	Number of Observations (%)	Labour Force Participants (Employment Rate %)	Average Monthly Employment Income (CNY)
**All women participants**	1274	573 (45)	1742
Women non-caregivers	750	370 (49)	1918
Women caregivers	524	203 (39)	1423
**Caregiving hours per week**			
At most 5 h	80 (15)	50 (63)	1816
Above 5 and at most 15 h	110 (21)	53 (48)	1420
Above 15 and at most 30 h	79 (15)	32 (41)	1478
Above 30 and at most 60 h	114 (22)	34 (30)	1262
Above 60 h	141 (27)	34 (24)	956
**Types of caregiver**			
Caregivers of only grandchildren	249 (48)	60 (24)	1085
Caregivers of only elders	215 (41)	129 (60)	1597
Caregivers of both dependents	60 (11)	232 (37)	1312
**All men participants**	2053	1538 (75)	2619
Men non-caregivers	1405	1072 (76)	2721
Men caregivers	648	466 (72)	2386
**Caregiving hours per week**			
At most 5 h	144 (22)	117 (81)	2918
Above 5 and at most 15 h	152 (23)	114 (75)	2183
Above 15 and at most 30 h	113 (17)	81 (72)	2677
Above 30 and at most 60 h	112 (17)	79 (71)	2004
Above 60 h	127 (20)	75 (59)	1956
**Types of caregiver**			
Caregivers of only grandchildren	288 (44)	175 (61)	2157
Caregivers of only elders	301 (47)	255 (85)	2536
Caregivers of both dependents	59 (9)	39 (66)	2444

Abbreviations: CNY, Chinese Yuan; h, hour.

**Table 2:** Comparing the characteristics of caregivers and non-caregivers among labour force–participating women and men.

	Women				Men			
Characteristics	All (*N* = 1274)	Non-Caregivers (*N* = 750)	Caregivers (*N* = 524)	*p*-Value	All (*N* = 2053)	Non-Caregivers (*N* = 1405)	Caregivers (*N* = 648)	*p*-Value
Labour force participants, N (%)	573 (45)	370 (49)	203 (38)		1538 (75)	1072 (76)	466 (72)	
Average monthly employment income, CNY, Mean (SD)	1742.21 (2101.68)	1917.54 (2448.66)	1422.65 (1181.42)	***	2619.35 (2690.37)	2720.61 (2773.56)	2386.41 (2475.63)	**
Average weekly caregiving hours, h, Mean (SD)	10.91 (25.28)	0	30.80 (34.55)		9.48 (24.05)	0	31.30 (35.03)	
Age, y, Mean (SD)	48.69 (3.00)	48.44 (2.96)	49.15 (3.03)	***	51.56 (4.50)	51.34 (4.50)	52.06 (4.47)	***
Currently married, N (%)	1198 (94)	713 (95)	487 (93)	>0.1	1991 (97)	1349 (96)	629 (97)	>0.1
Illiterate or with primary school education, N (%)	535 (42)	315 (42)	230 (44)	>0.1	698 (34)	492 (35)	214 (33)	>0.1
Middle school graduates, N (%)	369 (29)	218 (29)	142 (27)	>0.1	719 (35)	506 (36)	214 (33)	>0.1
High school graduates, N (%)	306 (24)	180 (24)	126 (24)	>0.1	513 (25)	337 (24)	175 (27)	>0.1
College and above, N (%)	64 (5)	37 (5)	26 (5)	>0.1	123 (6)	70 (5)	45 (7)	**
Having past-month outpatient visits or past-year inpatient stay, N (%)	229 (18)	113 (15)	131 (25)	***	308 (15)	211 (15)	104 (16)	>0.1
Rural residents, N (%)	459 (36)	278 (37)	178 (34)	>0.1	985 (48)	688 (49)	285 (44)	>0.1
Household size, Mean (SD)	3.53 (1.28)	3.39 (1.14)	3.78 (1.48)	***	3.83 (1.55)	3.71 (1.45)	4.09 (1.74)	***
Living with parents/in-law, N (%)	191 (15)	113 (15)	84 (16)	>0.1	349 (17)	211 (15)	130 (20)	**
Household yearly income from all sources, CNY, Mean (SD)	68,079.25 (176,634.81)	73,178.35 (216,493.74)	58,785.33 (50,946.63)	>0.1	59,947.80 (133,013.29)	62,285.82 (156,007.04)	54,569.34 (48,811.20)	>0.1
Working at a government or a state-owned organisation or firm, N (%)	280 (22)	158 (21)	121 (23)	>0.1	493 (24)	323 (23)	175 (27)	**
Working as a manager, N (%)	89 (7)	45 (6)	42 (8)	>0.1	(246) 12	155 (11)	91 (14)	>0.1

Continuous variables were compared using the 2-sample t-test (mean). Categorical variables were compared using the Chi-square test. Abbreviations: SD, standard deviation; y, year; h, hour; CNY, Chinese Yuan. * *p*-value < 0.1, ** *p*-value < 0.05, *** *p*-value < 0.01.

**Table 3:** Results of the Heckman selection model predicting log-transformed monthly employment income.

	Women	Men
Variables	(Caregiving Threshold at 63-h per Week)	(Caregiving Threshold at 15-h per Week)
Caregiving hours, per hour, CG	−0.0108 *	−0.00510
Discontinuity at the caregiving threshold, CG^	−1.359 *	0.184
Interaction between threshold and caregiving hours, CG*CG^	0.00384	−0.00000829
Age (per one-year increase)	−0.392	0.181
Age−squared	0.171	−0.277 *
Currently married	−0.763 **	0.694 ***
Middle school graduates	−0.0206	−0.0792
High school graduates	0.313 *	0.161 **
College graduates	1.455 **	0.416 ***
Having past-month outpatient visits or past-year inpatient admission	−0.788 **	−0.771 ***
Rural residence	0.196	0.740 ***
Household size (per one-person increase)	−0.162 **	−0.0623 ***
Living with parents/parents-in-law	0.441**	−0.0700
Yearly household income from all sources (log-transformed)	0.757 ***	0.743 ***
Working at the government or a state-owned organisation/firm	−0.326 **	−0.139 *
Working as a manager	0.798 ***	0.237 ***
Inverse Mills ratio	2.979 **	2.820 ***
Constant	13.14	−3.882
R-squared	0.169	0.196
F-statistic	6.644	21.85
Log-likelihood	−896.7	−2226
Joint significance test of the three caregiving variables (CG, CG^ and CG*CG^)	5.620 ***	2.525 *
Joint significance test of CG^ and CG*CG^	3.857 **	1.071

Abbreviations: h, hour. * *p*-value < 0.1, ** *p*-value < 0.05, *** *p*-value < 0.01.

**Table 4:** Results of separate Heckman selection modeling procedures for eldercare and childcare.

	**Eldercare**		**Childcare**	
	**Women**	**Men**	**Women**	**Men**
	**(*N* = 516)**	**(*N* = 1363)**	**(*N* = 449)**	**(*N* = 1286)**
**Variables**	**(Caregiving Threshold at 63-h per Week)**	**(Caregiving Threshold at 15-h per Week)**	**(Caregiving Threshold at 72-h per Week)**	**(Caregiving Threshold at 10-h per Week)**
Caregiving hours, per hour, CG	0.00243	0.000861	−0.0196 ***	0.00507
Discontinuity at the caregiving threshold, CG^	−2.713 **	0.272	1.965	−0.0680
Interaction between threshold and caregiving, CG*CG^	0.00916	−0.00134	−0.0134	−0.0106
Inverse Mills ratio	2.718 **	2.973 ***	2.584	3.27 ***
R-squared	0.172	0.205	0.160	0.188
F-statistic	6.074	20.35	4.827	17.28
Log-likelihood	−803.3	−2014	−708.8	−1905
Joint significance test of the three caregiving variables (CG, CG^ and CG*CG^)	7.077 ***	1.631	2.664 **	4.642 ***
Joint significance test of CG^ and CG*CG^	5.301 ***	1.149	1.473	0.184

Abbreviations: h, hour. * *p*-value < 0.1, ** *p*-value < 0.05, *** *p*-value < 0.01.

**Table 5:** Assessing the performance of the comprehensive model with four simpler models in predicting log-transformed monthly employment income.

	Women					Men				
Variables	CG as a Dummy Variable	CG as a Continuous Variable	Discontinuity-Only Model	Kink-Only Model	Our Model	CG as a Dummy Variable	CG as a Continuous Variable	Discontinuity-Only Model	Kink-Only Model	Our Model
Caregiver status (vs. non-caregivers)	−0.382 **					−0.0131				
Caregiving hours, per hour		−0.0173 ***	−0.00969 *		−0.0108 *		−0.00297 **	−0.00519 ***		−0.00510
Discontinuity at the threshold			−1.077 ***		−1.359 *			0.187		0.184
Interaction between threshold and caregiving				−0.0174 ***	0.00384				0.00309	−0.00000829
Inverse Mills ratio	2.117	2.762 **	2.953 **	2.823 **	2.979 **	2.792 ***	2.827 ***	2.830 ***	2.801 ***	2.820 ***
R-squared	0.144	0.160	0.169	0.161	0.169	0.195	0.195	0.196	0.195	0.196
F-statistic	6.230	7.093	7.060	6.653	6.644	24.54	24.63	23.12	23.05	21.85
Log-likelihood	−905.3	−899.7	−896.8	−899.6	−896.7	−2228	−2227	−2227	−2227	−2226
Likelihood-ratio test vs. our model	17.27 ***	5.98 **	0.17	5.76 **		3.12	1.97	1.41	2.31	

Abbreviations: h, hour. * *p*-value<0.1, ** *p*-value<0.05, *** *p*-value < 0.01.

## Data Availability

Publicly available datasets were analyzed in this study. These data can be found here: http://charls.pku.edu.cn/pages/data/2011-charls-wave1/en.html (accessed on 1 September 2020).

## Appendix A

**Table A1:** Results of an instrumental variables analysis to predict log-transformed monthly income among women and men.

	Women	Men
Variables	(Caregiving Threshold at 63 h per Week)	(Caregiving Threshold at 15 h per Week)
Caregiving hours, per hour, CG	0.416	0.0862
Discontinuity at the caregiving threshold, CG^	1.704	−6.835
Interaction between threshold and caregiving, CG*CG^	−0.430	0.0295
Age, per one-year increase	−0.932	−0.151
Age-squared	0.864	−0.184
Currently married	−	1.288
Middle school graduates	−0.535	−0.300
High school graduates	0.0769	0.283
College or above	−0.913	0.367 *
Having past-month outpatient visits or past-year inpatient admission	−0.527	−2.226
Rural area residents	−0.362	1.874
Household size, per one-person	−0.224	−0.143
Living with parents/parents-in-law	0.0676	−0.162
Yearly household income from all sources (log-transformed)	0.0517	1.559
Working at the government or a state-owned organization or firm	−0.438 *	−0.208 *
Employed as a manager	0.642	0.207 *
Inverse Mills ratio	−5.708	32.00
Constant	33.89 *	−9.400
Observations	536	1,528
R-squared	−0.358	−0.047
log-likelihood	−977.7	−2418
F-statistic	3.367	10.27
Joint significance test of the three caregiving variables (CG, CG^ and CG*CG^)	0.452 (*p*-value = 0.716)	0.859 (*p*-value = 0.462)
Joint significance test of CG^ and CG*CG^	0.658 (*p*-value = 0.518)	0.612 (*p*-value = 0.542)
**Validity tests of the instrumental variables**		
Kleibergen-Paap rk LM statistic (test of underidentification)	2.659 (*p*-value = 0.265)	6.545 (*p*-value = 0.0379)
Kleibergen-Paap rk Wald F statistic (weak IV test)	0.679	1.634
Endogeneity test statistic	4.742 (*p*-value = 0.192)	3.904 (*p*-value = 0.272)
Hansen J statistic (test of overidentification)	0.0965 (*p*-value = 0.756)	0.0943 (*p*-value = 0.759)

Abbreviations: h, hour. * *p*-value < 0.1, ** *p*-value < 0.05, *** *p*-value < 0.01.

**Table A2:** A secondary analysis on eldercare and childcare using the four instrumental variables.

	Eldercare		Childcare	
	Women	Men	Women	Men
	(*N* = 516)	(*N* = 1363)	(*N* = 449)	(*N* = 1286)
Variables	(Caregiving Threshold at 63 h per Week)	(Caregiving Threshold at 15 h per Week)	(Caregiving Threshold at 72 h per Week)	(Caregiving Threshold at 10 h per Week)
Caregiving hours, per hour, CG	−0.0809	6.400	−0.571	−2.026
Discontinuity at the caregiving threshold, CG^	9.176	−25.08	−1019	176.0
Interaction between threshold and caregiving, CG*CG^	−0.103	−6.363	8.793	0.407
Inverse Mills ratio	−4.513	−64.63	14.85	−10.00
R-squared	−1.051	−2.649	−134.868	−31.852
Log-likelihood	−950.1	−2961	−1851	−4284
F-statistic	2.952	2.008	0.390	0.452
Joint significance test of the three caregiving variables (CG, CG^ and CG*CG^)	0.645 (*p*-value = 0.586)	0.249 (*p*-value = 0.862)	0.00662 (*p*-value = 0.999)	0.0257 (*p*-value = 0.994)
Joint significance test of CG^ and CG*CG^	0.303 (*p*-value = 0.738)	0.270 (*p*-value = 0.764)	0.00547 (*p*-value = 0.995)	0.0326 (*p*-value = 0.968)
**Validity tests of the instrumental variables**				
Kleibergen-Paap rk LM statistic(test of under-identification)	4.786 (*p*-value = 0.310)	1.811 (*p*-value = 0.770)	9.610 (*p*-value = 0.383)	8.947 (*p*-value = 0.442)
Kleibergen-Paap rk Wald F statistic (weak IV test)	0.740	0.298	1.112	0.881
Endogeneity test statistic	5.839 (*p*-value = 0.120)	5.279 (*p*-value = 0.152)	4.902 (*p*-value = 0.179)	2.945 (*p*-value = 0.400)
Hansen J statistic (test of overidentification)	1.156 (*p*-value = 0.763)	0.530 (*p*-value = 0.912)	1.962 (*p*-value = 0.982)	1.499 (*p*-value = 0.993)

Abbreviations: h, hour. * *p*-value < 0.1, ** *p*-value < 0.05, *** *p*-value < 0.01.

**Table A3:** Results of the Heckman selection model predicting log-transformed monthly employment income using the pooled sample of women and men.

Variables	Pooled Sample (Caregiving Threshold at 72 h per week)
Caregiving hours before the threshold, per hour, CG	−0.00743 ***
Discontinuity at the caregiving threshold, CG^	0.921 *
Interaction between threshold and caregiving, CG*CG^	−0.00564
Age (per one-year increase)	−0.150
Age-squared	0.0643
Male (vs. female)	1.381 ***
Currently married	0.182
Middle school graduates	0.00667
High school graduates	0.225 ***
College graduates	0.648 ***
Having past-month outpatient visits or past-year inpatient admission	−0.515 ***
Rural residence	0.413 ***
Household size (per one-person increase)	−0.0673 ***
Living with parents/parents-in-law	0.0225
Yearly household income from all sources (log-transformed)	0.597 ***
Working at the government or a state-owned organisation/firm	−0.175 ***
Working as a manager	0.339 ***
Inverse Mills ratio	1.764 ***
Constant	5.128
Observations	2111
R-squared	0.189
F-statistic	27.02
Log-likelihood	−3160
Joint significance test of the three caregiving variables (CG, CG^ and CG*CG^)	8.918 (*p*-value < 0.001)
Joint significance test of CG^ and CG*CG^	2.142 (*p*-value = 0.118)

Abbreviations: h, hour. * *p*-value < 0.1, ** *p*-value < 0.05, *** *p*-value < 0.01.

**Table A4:** Results of an instrumental variables analysis conducted on the pooled sample.

Variables	Pooled Sample (Caregiving Threshold at 72 h per Week)
Caregiving hours before the threshold, per hour, CG	0.611
Discontinuity at the caregiving threshold, CG^	−66.59
Interaction between threshold and caregiving, CG*CG^	−0.0579
Age (per one-year increase)	0.137
Age-squared	0.242
Male (vs. female)	−6.601
Currently married	−1.289
Middle school graduates	−0.429
High school graduates	−1.169
College graduates	−2.258
Having past-month outpatient visits or past-year inpatient admission	2.732
Rural area residents	−1.355
Household size, per one-person	−0.286
Living with parents/parents-in-law	−1.899
Yearly household income from all sources (log-transformed)	−1.267
Working at the government or a state-owned organization or firm	−0.368
Employed as a manager	0.284
Inverse Mills ratio	−53.67
Constant	34.58
Observations	2064
R-squared	−1.811
Log-likelihood	−4380
F-statistic	5.312
Joint significance test of the three caregiving variables (CG, CG^ and CG*CG^)	0.249 (*p*-value = 0.862)
Joint significance test of CG^ and CG*CG^	0.136 (*p*-value = 0.873)
Tests establishing the validity of the instruments	
Kleibergen-Paap rk LM statistic (under-identification)	2.152 (*p*-value = 0.341)
Kleibergen-Paap rk Wald F statistic (weak IV test)	0.536
Endogeneity test statistic	3.580 (*p*-value = 0.310)
Hansen J statistic (overidentification)	0.469 (*p*-value = 0.493)

Abbreviations: h, hour. * *p*-value < 0.1, ** *p*-value < 0.05, *** *p*-value < 0.01.

**Table A5:** Subgroup analysis on women using the Heckman procedure and assuming a weekly caregiving threshold of 63 h.

	Living with Parents or Parents-in-Law		Self-Employed		Hukou Status		Educational Status		Household Income		Hukou Region (Economic Regions)			Hukou Region (City Tiers)	
Variables	No	Yes	No	Yes	Rural	Urban	Below Middle School	Middle School or above	Below the Median	At Least the Median	East	Middle	West	Tier 2 or above	Below Tier 2
Caregiving hours, CG	−0.0154 *	0.00318	−0.00860	−0.0308 *	−0.00663	−0.0138	−0.00640	−0.0364 ***	−0.0107	−0.0191 *	−0.00898	−0.0194	−0.0186	−0.00477	−0.0185 *
Discontinuity, CG^	−1.516	−0.821	−1.839 *	−0.959	−1.570*	−0.836	−0.441	−3.132 **	−1.450	−1.834	−1.472 *	−2.560	−3.758	−0.743	−1.789 *
Interaction, CG*CG^	0.000673	0.0167	0.00412	0.0223	0.0111	−0.0179	−0.00909	0.0188	−0.00710	0.0168	0.0159	0.00719	0.0358	−0.00499	0.00865
Inverse Mill’s ratio	4.581 **	−3.535	2.833	4.459	1.677	4.073	2.105	11.32 ***	4.555	4.041	0.113	5.617 *	7.181	2.119	4.796 **
Observations	486	87	463	110	367	206	242	331	270	377	295	165	113	213	360
R-squared	0.171	0.478	0.156	0.447	0.187	0.255	0.256	0.196	0.115	0.131	0.250	0.263	0.208	0.181	0.206
F-statistic	6.064	4.003	4.839	5.479	5.035	4.040	5.577	5.508	2.062	3.382	5.419	3.078	1.465	2.530	5.232
log-likelihood	−781.3	−84.09	−737.9	−135.7	−527.4	−343.1	−337.7	−534.2	−447	−537.4	−397.2	−264.7	−192.1	−345.4	−539.5
Joint sig of three CG variables	8.171 ***	1.227	6.073 ***	2.182*	1.794	5.714 ***	3.489 **	10.08 ***	2.464 *	2.823 **	1.738	4.732 ***	0.493	1.500	6.073 ***
Joint sig of CG^ and CG*CG^	5.242 ***	0.726	5.704 ***	1.005	2.465 *	3.894 **	2.708 *	4.437 **	3.621 **	1.913	2.134	2.811 *	0.360	1.498	3.090 **

* *p*-value < 0.1, ** *p*-value < 0.05, *** *p*-value < 0.01.

**Table A6:** Subgroup analysis on men using the Heckman procedure and assuming a weekly caregiving threshold of 15 h.

	Living with Parents or Parents-in-Law		Self-Employed		Hukou Status		Educational Status		Household Income		Hukou Region (Economic Regions)			Hukou Region (City Tiers)	
Variables	No	Yes	No	Yes	Rural	Urban	Below Middle School	Middle School or above	Below the Median	At Least the Median	East	Middle	West	Tier 2 or above	Below tier 2
Caregiving hours, CG	0.00183	−0.0309	−0.00209	−0.0161	0.00834	−0.0199	0.0338	−0.0232	−0.00816	−0.0217	−0.0128	−0.0146	0.0223	−0.0104	−0.00232
Discontinuity, CG^	0.240	−0.00435	0.255	−0.307	0.178	0.231	0.0827	0.218	0.0231	−0.00432	0.320	0.0333	0.236	0.233	0.147
Interaction, CG*CG^	−0.00800	0.0285	−0.00373	0.0186	−0.0139	0.0134	−0.0423	0.0212	0.00927	0.0182	0.00511	0.0123	−0.0289	0.00364	−0.00232
Inverse Mill’s ratio	3.120 ***	2.191 **	2.691 ***	1.546	3.248 ***	2.518 *	2.155 **	2.339 ***	−0.0131	0.544	3.187 ***	2.040 *	3.089 *	2.784 ***	2.913 ***
Observations	1280	258	1245	293	1051	487	530	1008	789	871	666	528	344	476	1062
R-squared	0.186	0.301	0.159	0.456	0.210	0.227	0.118	0.207	0.041	0.095	0.179	0.272	0.219	0.207	0.203
F-statistic	18.06	6.496	13.68	15.51	17.17	8.615	4.945	18.56	2.038	5.574	8.296	11.21	5.377	7.017	15.60
Log-likelihood	−1846	−368.5	−1826	−361.9	−1391	−785.3	−774.5	−1463	−1242	−1226	−903.5	−705.2	−563.8	−619.4	−1587
Joint sig of three CG variables	2.380 *	0.932	2.359 *	0.720	3.049 **	0.923	2.069	1.980	0.183	1.662	2.080	0.668	0.732	1.055	1.653
Joint sig of CG^ and CG*CG^	1.290	0.924	1.560	0.736	1.254	0.711	1.815	2.897 *	0.194	0.854	1.709	0.348	0.615	0.709	0.401

* *p*-value < 0.1, ** *p*-value < 0.05, *** *p*-value < 0.01.
